# Impact of Type and Enzymatic/High Pressure Treatment of Milk on the Quality and Bio-Functional Profile of Yoghurt

**DOI:** 10.3390/foods9010049

**Published:** 2020-01-04

**Authors:** Maria Tsevdou, Georgios Theodorou, Sofia Pantelaiou, Artemis Chatzigeorgiou, Ioannis Politis, Petros Taoukis

**Affiliations:** 1 Laboratory of Food Chemistry and Technology, School of Chemical Engineering, National Technical University of Athens, 5 Heroon Polytechniou Str., 157 80 Athens, Greece; mtsevdou@chemeng.ntua.gr (M.T.); sofiapantelaiou@gmail.com (S.P.); taoukis@chemeng.ntua.gr (P.T.); 2 Department of Animal Science, Agricultural University of Athens, 75 Iera Odos Str., 118 55 Athens, Greece; gtheod@aua.gr; 3 DELTA FOODS S.A., Department of Research and Development, Aghios Stefanos Plant, 145 65 Athens, Greece; arthat@delta.gr

**Keywords:** high pressure, transglutaminase, low-fat yoghurt, quality characteristics, anti-hypertensive activity, immunomodulatory properties

## Abstract

The objective of the present study was to investigate the effect of the high pressure (HP) processing and transglutaminase (TGase) treatment of bovine (cow) or ovine (sheep) milk, when applied individually or sequentially, on the quality parameters and anti-hypertensive and immunomodulatory properties of yoghurt. Low-fat (2% w/w) bovine or ovine milk samples were used. Results showed that HP treatment of milk led to acid gels with equivalent quality attributes to thermal treatment, with the more representative attributes being whey separation and firmness, which ranged from 47.5% to 49.8% and 23.8% to 32.2% for bovine and ovine yoghurt, respectively, and 74.3–89.0 g and 219–220 g for bovine and ovine yoghurt, respectively. On the other hand, TGase treatment of milk, solely or more effectively following HP processing, resulted in the improvement of the textural attributes of yoghurt and reduced whey separation, regardless of milk type, exhibiting values of 32.9% and 8.7% for the whey separation of bovine and ovine yoghurt, respectively, and 333 g and 548 g for the firmness of bovine and ovine yoghurt, respectively. The HP processing and TGase treatment of milk led to the preservation or improvement of the anti-hypertensive activity of the samples, especially in the case in which ovine milk was used, with Inhibitory activity of Angiotensin Converting Enzyme (IACE) values of 76.9% and 88.5% for bovine and ovine yoghurt, respectively. The expression of pro-inflammatory genes decreased and that of anti-inflammatory genes increased in the case of samples from HP-processed and/or TGase-treated milk as compared to the corresponding expressions for samples from thermally treated milk. Thus, it can be stated that, apart from the quality improvement, HP processing and TGase treatment of milk may lead to the enhancement of the bio-functional properties of low-fat yoghurt made from either bovine or ovine milk.

## 1. Introduction

Over the last decades, in order for consumers’ demands for health-promoting food products to be satisfied, a large amount of research has been conducted in functional foods. These products include non-fat or low-fat food products and others that are fortified with natural functional ingredients, or in which these ingredients are activated. With respect to dairy products, a great number of non-fat and low-fat yoghurt and yoghurt-like functional products have been introduced in the global market. However, during their production, major issues such as the weak texture and whey separation phenomena caused by the low total solids content have to be addressed [1]. In this context, the high pressure (HP) processing of milk and the cross-linking of milk proteins via transglutaminase (TGase) are proposed as alternatives to the costly protein fortification or addition of stabilizing agents in milk [2]. These technologies are reported to improve the yoghurt-making properties of milk and consequently the whey separation phenomena, post-acidification, textural and rheological attributes, sensory characteristics, and flavor evolution and release in either set or stirred yoghurt [2,3,4,5,6,7,8]. However, to the best of our knowledge, these technologies have been studied mainly in bovine milk sources, while little or almost no research has been conducted in ovine milk samples [9].

Among milk components, proteins can be considered as extremely important due to their nutritional value along with their technological and bio-functional properties. Their nutritional value is related to their contribution to satisfying the nitrogen and amino acid requirements for the growth and maintenance of the human body [10]. Additionally, milk proteins can act as precursors for bioactive peptides, usually ranging in size from 2 to 23 amino acids; these peptides are inactive within the native protein sequence, but may be released by enzymatic hydrolysis or fermentation through the action of digestive proteases or proteolytic starter cultures, respectively. Once they are released, they exhibit biological activity; i.e., antimicrobial, antihypertensive, antithrombotic and immunomodulatory activity [11,12].

Anti-hypertensive peptides are the most studied group of bio-functional peptides since they are related to cardiovascular diseases (CVDs), which are the most important cause of death, representing 31% of all deaths globally in 2016 [13]. Both caseins and whey proteins seem to be a rich source of peptides with anti-hypertensive activity [14]. In particular, casein-derived peptides including the fragments 23–24, 23–27 and 194–199 of αs1-casein and 63–68, 177–183, 191–193 and 193–202 of β-casein are reported to inhibit in vitro angiotensin converting enzyme (ACE) activity. Additionally, with regard to peptides presenting immunomodulatory action, the tripeptide Leu-Leu-Tyr and the hexapeptide Pro-Gly-Pro-Ileu-Pro-Asp of milk proteins are reported to present anti-inflammatory activity [12,15,16,17,18,19]. It has been previously shown that yoghurt possesses both anti-hypertensive [20] and immunomodulatory properties [21]. However, the effect of the implementation of the alternative treatment of milk on these properties of yoghurt has not yet been studied. The expression of a wide panel of immune-related genes, such as inducible nitric oxide synthetase (NOS2), cyclooxygenase-2 (PTGS2), transforming growth factor beta 1 (TGFB1) and interleukins 1, 10 and 12 (IL1B, IL10 and IL12B)—known to be related to inflammatory response and involved in a variety of cellular activities, including cell signaling, proliferation, differentiation, activation, etc.—was assessed in order to evaluate the immunomodulatory activity of yoghurt. The ability of fermented dairy products to modulate the expression of the above-mentioned genes in vitro can serve as strong evidence of immunomodulatory activity since their protein products play a crucial role in several physiological and pathological conditions such as simple pain and hypersensitivity, acute and chronic inflammation, multiple sclerosis, tumor growth, etc. [12,22,23,24,25].

Based on the aforementioned scientific gaps, the aim of this study was (a) to investigate the effect of the HP processing and TGase treatment of low-fat milk on the technological attributes and bio-functional properties of set yoghurt made from ovine milk, as evaluated through its physicochemical, textural and structural characteristics and its anti-hypertensive activity and immunomodulatory properties, respectively, and (b) to compare the results of these processes with the corresponding properties of yoghurt samples made from bovine milk.

## 2. Materials and Methods

### 2.1. Milk Treatment and Yoghurt Sample Preparation

Raw low-fat bovine and ovine milk samples, with a standardized fat content of 2.09% (±0.14) w/w, were used in our experiments. The protein content of milk was estimated at 3.10% w/w (±0.00) for bovine and 5.53% w/w (±0.18) for ovine milk samples. Four different bovine and corresponding ovine yoghurt samples (Table 1) were prepared as described in Tsevdou et al. [2], with the following modifications:

- Milk homogenization was performed in a single stage process at 150 bar;
- Thermal treatment of milk was conducted at 95 °C for 5 min;
- TGase (ACTIVA YG, Ajinomoto, DE) was inoculated at an enzyme concentration of 2.0 U·g^−1^ protein (reference activity: 100 U·g^−1^);
- The commercial starter culture used was Yo-Mix (Danisco, DK) prepared as a 1:4 (w/w) dilution in commercial UHT (Ultra-High-Temperature) skim milk.

### 2.2. Fermentation Kinetics

A high precision pH meter (AMEL 338, AMEL Instruments, Milano, Italy) was used for pH measurements during the fermentation procedure. The pH of yoghurt samples during the fermentation process was continuously monitored using a 12 mm glass electrode (HI1131B, Hanna Instruments, Winsocket, RI, USA). The pH was recorded every 20 min at the beginning and every 10 min after 2.5 h of fermentation, until the end point of fermentation (pH 4.75). The pH values were plotted against time, and the parameters describing the fermentation kinetics were determined by fitting the data to the modified Gompertz equation (Equation (1)), as previously proposed by de Brabandere and de Baerdemaeker [26]:

(1) $$pH=pH_{0}+(pH_{\infty }-pH_{0})\exp\left\{-\exp\left[\frac{\mu e}{\left(pH_{\infty }-pH_{0}\right)}(\lambda -t)\right]+1\right\}$$

where *pH*∞ and *pH*_0_ show the final (end point) and initial pH values respectively, *μ* shows the maximal acidification (pH drop) rate expressed in pH·min^−1^, and *λ* is the duration of the lag phase (min).

### 2.3. Study of the Quality Characteristics of Yoghurt

The microbiological quality of the prepared samples was tested in weekly intervals with respect to total viable counts (TVC), yeasts and molds, and starter culture growth, as described in Tsevdou et al. [2]. Briefly, 10-fold serial dilutions of yoghurt samples were either spread or pour-plated in the appropriate growth media in Petri dishes for the enumeration of different microorganisms. Total viable counts were enumerated in Plate Count Agar (Merck, DE) after incubation at 25 °C for 72 h under aerobic conditions. Viable yeast and molds were enumerated on Rose Bengal Chloramphenicol (RBC) Agar (Merck, Germany) after incubation at 25 °C for 72 h under aerobic conditions. *Streptococcus thermophilus* was enumerated on M17 Agar (Merck, DE) after incubation at 37 °C for 24 h under aerobic conditions. *Lactobacillus bulgaricus* was enumerated in De Man–Rogosa–Sharpe (MRS) Agar with a modified pH value at 4.58 (Merck, DE) after incubation at 45 °C for 72 h in anaerobic jars with an Anaerocult A catalyst (Merck, DE).

The acidity of yoghurt samples was measured using a pH meter (AMEL 338, AMEL Instruments, IT) and by the titration of a 1:1 mix of yoghurt/deaerated-deionized water with 0.1 N NaOH using phenolphthalein as an indicator, and expressed as % lactic acid [27].

The susceptibility of yoghurt to whey separation was determined using a drainage method and was expressed as the grams of separated whey from 100 g of sample after incubation at 4 °C for 3 h. Briefly, 100 g of yoghurt was transferred to a funnel with Whatman paper #1 placed on a conical flask. The flask was stored at 4 °C and the amount of eliminated serum was weighted after 3 h of storage.

Texture analysis was performed using a TA-XT Plus texture analyzer (Stable Micro Systems, Surrey, UK) and the microstructure of the prepared acid gels was examined with scanning electron microscopy (SEM), as previously described in Tsevdou et al. [2]. Briefly, for texture analysis, samples were tempered at 10 °C before testing, and then they were subjected to a double compression test using a clear acrylic cylinder probe TA3/1000 of 25.4 mm in diameter and 35 mm in length (Brookfield Viscometers Ltd., Harlow Essex, UK). For SEM analysis, samples were freeze-dried using a laboratory scale freeze-drying unit (Alpha 1-4LDplus, CHRIST, Germany) and then gold–palladium-coated in vacuum using a sputtering device (Polaron 5100). The microstructure was examined with a FEI Quanta 200 (FEI Company, Hillsborough, OR, USA) scanning electron microscope using a large-field detector (LFD) operating at 25 kV.

### 2.4. Study of the Bio-Functional Properties of Yoghurt

#### 2.4.1. Preparation of Water-Soluble Extracts (WSEs)

Water-soluble extracts (WSEs) were obtained from all samples after 3 and 42 days of storage using the method proposed by Kuchroo & Fox [28]. Briefly, a mixture of 1:2 yoghurt/deionized water was prepared and homogenized in a Bag Stomacher (BagMixer Interscience, FR) for 10 min, followed by incubation in a water-bath of 45 °C for 1 h. The incubated samples were then centrifuged (Heraeus Megafuge 16R, Thermo Fischer Scientific, OR, USA) at 3000× *g* and 20 °C for 30 min. The supernatant was collected, vacuum-filtered and stored in a freezer until the analysis. According to the above procedure, one third (ca. 34%) of the total water-soluble nitrogen was extracted.

#### 2.4.2. Determination of ACE-Inhibitory Activity

ACE-inhibitory activity was determined using the method proposed by Nakamura et al. [29], as modified by Donkor et al. [20]. Each assay mixture (250 μL) contained the following components at the indicated final concentrations: (a) 180 μL of a hippuryl-L-histidyl-L-leucine solution (5 mM) in sodium borate (100 mM) (pH 8.3), buffer-treated with 300 nM NaCl; (b) 50 μL of WSE or peak material or water as a blank; (c) 20 μL of a 2 mU ACE (from rabbit lung, Sigma-Aldrich, St. Louis, MO, USA) aqueous solution. The mixture was incubated at 37 °C for 90 min, and the reaction was stopped with 250 μL of 1 N HCl. The produced hippuric acid was extracted with 1.7 mL of ethyl acetate. In order to separate the organic phase, samples were centrifuged at 1000 rpm and 15 °C for 10 min. Samples were then heat-evaporated at 100 °C for 15 min, re-dissolved in 1 mL of distilled water and measured spectrophotometrically at 228 nm (SPECTROStar Nano, BMGLabtech, DE). The ACE-inhibitory (IACE) activity was expressed as the percentage according to the following equation:

(2) $$ACE-inhibitory\;activity^{}(\%)=\left[1-\frac{C-D}{A-B}\right]\cdot 100$$

where Α is the absorbance of the ACE–buffer solution, Β is the absorbance of the buffer solution, C is the absorbance of ACE in WSE, and D is the absorbance of the WSE–buffer solution.

#### 2.4.3. Determination of Immunomodulatory Activity

The immunomodulatory activity of WSEs was evaluated by measuring their ability to modulate the expression of the pro-inflammatory genes IL1B, IL12B, NOS2 and PTGS2, and of the anti-inflammatory genes IL10 and TGFB1 by ovine monocytes. Ovine monocyte isolation, treatment with WSEs, RNA isolation and subsequent enzymatic reactions were performed as described by Theodorou and Politis [30].

### 2.5. Statistical Analysis

The data represent the means with their standard deviation of two independent experiments. Within each experiment, each treatment was performed in triplicate. A factorial ANOVA was applied for the determination of the main effects of the investigated factors (milk type, milk treatment, storage time) and their interactions on the experimental data. Duncan’s multiple range test was used to separate the means of data when significant differences (*p* < 0.05) were observed. All statistical analyses were performed using Statistica^®^ release 7 software (StatSoft Inc., Tulsa, OK, USA).

## 3. Results and Discussion

### 3.1. Effect of Milk Type and Applied Treatment on the Fermentation Kinetics of Milk

The fermentation kinetic parameters (lag phase duration, λ, and maximal acidification rate, μ) were estimated with a modified Gompertz model (Equation (1)) and are presented in Table 2. The enzymatic treatment with TGase of bovine milk led to a significant (*p* < 0.001) reduction in the lag phase duration, whereas the HP treatment of both bovine and ovine milk led to a slight—yet significant (*p* < 0.01)—increase in the lag phase duration. However, the sequential application of the HP and TGase treatment of milk led to a pronounced reduction of the duration of the lag phase compared to samples from either thermally or HP-treated milk. Additionally, the milk type, which is directly related to compositional differences, had a significant (*p* < 0.001) effect on lag phase duration, as previously reported [31,32]. In particular, ovine yoghurt samples exhibited a decreased lag phase as compared to bovine samples, which might be related to the increased protein content of ovine milk and thus to the higher availability of free amino acids, which is related to the proteolytic activity of the starter culture [31].

In both bovine and ovine milk, TGase treatment led to a significant (*p* < 0.05) increase in the maximal acidification rate when HP treatment was previously applied. This behavior might be correlated with extended cross-linking among milk proteins and the increased availability of free amino acids for the proteolytic action of the starter culture microorganisms [33]. The HP treatment of milk also led to a significant (*p* < 0.01) increase in the maximal acidification rate, as compared to the corresponding value of thermally treated milk samples, regardless of the milk type, as a result of the protein unfolding and refolding induced by HP treatment [9,33].

### 3.2. Effect of Milk Type, Applied Treatment and Storage Time on the Quality Attributes of Yoghurt

According to the microbiological analyses, all samples exhibited growth below the detection limit (<2 logCFU/g) for yeasts and molds, whereas the total counts of the starter culture (*Streptococcus thermophilus* and *Lactobacillus bulgaricus*) remained above the acceptable limit of 7 logCFU/g, during the 42 days of storage, as also reported in previous studies [2,8].

With regard to acidity, it was found to be significantly (*p* < 0.01) dependent on milk type, TGase treatment of milk and storage time for all tested samples. In particular, in the case of bovine yoghurt samples, the initial pH ranged from 4.18–4.51 for samples from alternatively treated milk compared to 4.48 for samples from thermally treated milk (Table 3), and these values decreased to 4.18–4.21 and 4.12, respectively, after 42 days (data not shown). As has been also reported in previous studies [34,35], this is probably related to the slow acidity development induced by the delayed multiplication of the starter culture microorganisms. Similar results were observed in the case of ovine yoghurt samples, with the difference that, in general, ovine samples exhibited higher pH values than bovine samples, indicating that the type of milk, and consequently the milk composition, plays an important role in the development of quality attributes—e.g., post-acidification—in fermented products [31].

The pre-treatment of milk (thermally or HP), TGase treatment, milk type and storage time all had a significant (*p* < 0.001) effect on whey separation percentages. The HP treatment of milk led to either a similar or slight—yet significant (*p* < 0.05)—increase in whey separation as compared to samples from thermally treated milk for either bovine or ovine yoghurts (Table 3). The subsequent thermal treatment and enzymatic cross-linking of milk proteins resulted in a significant (*p <* 0.001) decrease in whey percentages after 3 days of production as compared to samples from either thermally or HP-treated milk, regardless of the milk type. The decrease in syneresis, which is associated with an improvement in the protein network of the studied yoghurt samples [34], was even more pronounced in the case in which TGase was combined with a prior step of HP treatment. As a result, yoghurt samples from HP–TGase-treated milk exhibited the lowest percentages of whey separation (with statistical mean percentages of 32.9% and 8.76% for bovine and ovine samples, respectively). Similar results have been also previously reported by Anema et al. [36] and Tsevdou et al. [2], who identified that both simultaneous TGase treatment under HP conditions and/or the subsequent HP processing and TGase treatment of bovine milk are capable of causing the extensive denaturation of whey proteins and dissociation of micelles, reforming new intra- and inter-molecular cross-links and leading to strengthened networks with contiguous protein molecules and thus with reduced water permeability. With regard to the effect of milk type on the whey separation, previous studies in goat, sheep and cow milk showed that yoghurt gels prepared from ovine milk exhibit the lowest percentages of whey separation compared to yoghurts from either goat or bovine milk due to the different chemical composition of these milks regarding their proportion of the four major caseins [31].

The major textural attributes (firmness and adhesiveness) of both bovine and ovine yoghurt samples were found to be significantly (*p* < 0.001) dependent on all designed parameters—milk pre-treatment, TGase treatment, storage time, as well as milk type. The TGase treatment of milk, when applied individually—or to a greater extent subsequently to HP treatment—resulted in the highest values of firmness for all tested samples (Table 3). However, the HP treatment of milk led to a slight, but not significant, decrease in the firmness values of bovine samples as compared to those of samples prepared from thermally treated milk. Both the treatment of milk and milk type had effects in the same manner as in the case of whey separation on the firmness of the prepared yoghurt samples, which is related to both the protein denaturation and further cross-linking of the milk proteins and the different protein content of bovine and ovine milk. As expected, storage time had a significant (*p* < 0.001) effect on firmness for all tested samples—regardless of milk composition, milk type or the applied treatment in milk—as a result of the continuous starter culture multiplication during storage and thus the strengthening of the coagulum and an increase in firmness values.

Concerning the parameter of adhesiveness, this was found to be significantly (*p* < 0.001) dependent on all experimental designing factors, with the exception of storage time (Table 3). The application of the HP treatment of bovine milk led to samples with high absolute values of adhesiveness (with a statistical mean value of −44.6 g·s), especially when combined with a subsequent step of TGase treatment (with a statistical mean value of −121 g·s). This observation had been previously correlated with a smoother and creamier sensorial perception of those products as compared to products prepared from thermally treated milk [37]. Yoghurt samples prepared from ovine TGase-treated milk exhibited the lowest absolute value of adhesiveness, which was previously correlated with a more gel-like sensorial perception of these products [2]. However, samples prepared from HP–TGase-treated milk exhibited lower value of adhesiveness (with a statistical mean value of −94.3 g·s) compared to samples from either thermally (with a statistical mean value of −152 g·s) or HP-treated (with a statistical mean value of −96.1 g·s) milk, but these values were higher than those of samples from TGase milk (with a statistical mean value of −80.1 g·s), suggesting that the HP treatment of milk maintains the smooth and creamy structure of the coagulum, while the combination with the TGase treatment of milk may also lead to the improvement of the protein network of acid gels.

**Figure 1:**
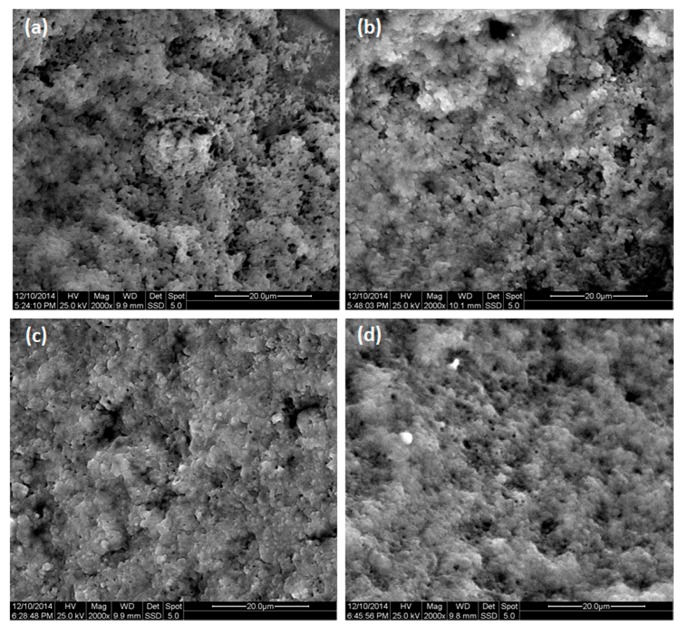
Scanning electron micrographs (× 2000) of bovine yoghurt prepared from thermally treated (**a**), thermal–TGase-treated (**b**), HP-treated (**c**) and HP–TGase-treated milk (**d**), respectively.

In order to confirm our observations concerning the improvement of the protein network in our yoghurt samples prepared from alternatively treated milk, samples prepared from bovine milk were analyzed by scanning electron microscopy (Figure 1a–d). As illustrated in Figure 1a–b, the observed protein network of TGase-treated samples was more compact than that of thermally treated samples, which is related to the smaller porosity and strengthening of the protein network [34,38]. Similar improvements were observed in the case in which HP treatment was applied (Figure 1c). In the case of yoghurt prepared from HP–TGase treated milk, the samples exhibited an even tighter protein network as compared to all other samples (Figure 1d), revealing more continuous cross-links between protein molecules and fewer and smaller lacunae (depicted as black areas on SEM images) in comparison with samples prepared from HP or TGase-treated milk, thus supporting the hypothesis regarding the synergistic effect of the HP and subsequent TGase treatment of milk on its yoghurt-making properties.

### 3.3. Effect of Milk Type, Applied Treatment and Storage Time on the Anti-Hypertensive Activity of Yoghurt

**Figure 2:**
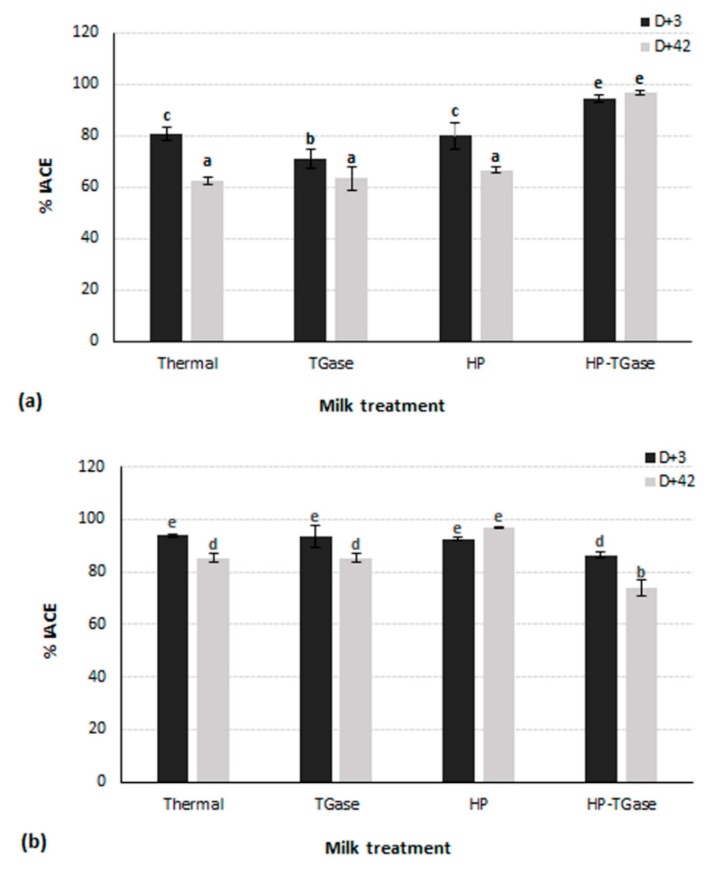
Anti-hypertensive properties of bovine (**a**) and ovine (**b**) water-soluble enzymes (WSEs), as expressed though the % angiotensin converting enzyme inhibitory activity (% IACE). Different letters among bars indicate significant differences (*p* < 0.05) between tested samples according to Duncan’s mean values post hoc comparison test.

The anti-hypertensive activity of the prepared yoghurt samples (as expressed through % IACE) was found to be significantly (*p* < 0.001) dependent on all designed parameters—milk pre-treatment, TGase treatment, storage time, as well as milk type (Figure 2).

Ovine WSEs samples exhibited significantly (*p* < 0.001) higher %IACE percentages than the bovine WSEs samples (with statistical mean percentages of 76.9% and 88.5% for bovine and ovine WSEs, respectively), which is in agreement with previous observations on commercially available fermented products made from thermally treated bovine or ovine milk, suggesting that the proteolysis of ovine milk probably leads to the release of peptides which are more capable of inhibiting the ACE-system [21,39]. Regarding the HP treatment of milk, it led to a significant (*p* < 0.001) increase in %IACE as compared to the corresponding percentage of samples from thermally treated milk, especially when combined with a subsequent enzyme treatment (with statistical mean percentages of 78.4%, 83.9% and 87.9% for samples from thermally, HP and HP–TGase-treated milk, respectively). Concerning the effect of storage period on the anti-hypertensive activity, it was observed that there is a noteworthy synergistic effect of both storage time and milk type, as samples prepared from different milk samples behaved differently, with samples from HP and HP–TGase-treated milk showing the best preservation or even enhancement of anti-hypertensive activity of yoghurt samples prepared from ovine and bovine milk, respectively. To date, there have been no studies on the effect of either the HP or TGase treatment of milk on the anti-hypertensive activity of fermented milk products, and therefore, a more detailed analysis of specific peptidic fractions with ACE-inhibitory activity may reveal important information regarding the mechanism of these effects on the release of bioactive peptides.

### 3.4. Effect of Milk Type, Applied Treatment and Storage Time on the Immunomodulatory Properties of Yoghurt

The immunomodulatory properties of WSEs were found to be significantly (*p <* 0.001) dependent on milk type and milk pre-treatment, whereas storage time did not affect the relative expression of the tested genes by ovine monocytes, with the exception of the pro-inflammatory IL12B (Table 4). For all tested genes, their expression by monocytes was significantly (*p <* 0.001) higher when cells were treated with ovine WSEs than when treated with bovine WSEs (Table 4).

For all tested genes, their expressions were found not to be affected by the applied processing of the milk, with the exception of TGFB1, where HP—applied individually or in combination with a subsequent step of enzymatic treatment—led to a significant (*p <* 0.001) decrease in expression (Table 4). Additionally, the fact that the expression of the pro-inflammatory NOS2 was not affected by the applied treatment in milk is important, as this gene is associated with the defense mechanism in humans (e.g., attack by parasites, bacterial infections, tumors growth, etc.) and also plays a critical role in many diseases with an autoimmune etiology [40]. With regard to IL10 gene expression, although no differences were observed among samples after 3 days of production, at the end of the storage period (42 days), its relative expression was significantly (*p <* 0.001) higher as compared to its initial expression only in the case in which ovine milk was subjected to both HP and TGase treatment.

Concerning the effect of ovine WSEs on the relative expression of pro-inflammatory genes by ovine monocytes, it was observed that the treatment of monocytes with WSEs from yoghurts produced with TGase-treated milk led to a significant (*p <* 0.001) decrease in both IL12B and IL1B expressions (Table 4), whereas treatment with WSEs from yoghurts produced with HP treatment of milk led to a significant (*p <* 0.05) decrease in NOS2. In the case of PTGS2, no differences were detected among samples. Similar results have been previously reported by Cermẽno et al. [41], who found that the addition of TGase prior to and after the hydrolysis of sodium caseinate showed a significant decrease in the release of the pro-inflammatory gene IL-6.

Considering that the levels of IL12B and IL1B are associated with auto-inflammatory syndromes [23] and diseases of the nervous system [22], respectively, their expression is required to be maintained at low levels. Regarding the relative expression of the tested anti-inflammatory genes by ovine monocytes, it was observed that each gene was influenced differently by the applied treatment in milk. The HP processing of milk led to a significant (*p* < 0.05) increase in TGFB1 expression (Table 4, with a statistical mean value of 3.058), whereas it led to a significant (*p* < 0.05) decrease in IL10 expression (Table 4, with a statistical mean value of 2.491), as compared to the corresponding values of monocytes treated with WSEs from thermally treated milk samples (with statistical mean values of 2.448 and 3.052 for TGFB1 and IL10, respectively). The enhancement of TGFB1 expression is desirable as it plays an important role in controlling the immune system and has a key role in the resolution of inflammation [42]. It is also worth noting that both bovine and ovine WSE, affected the expression of pro- and anti-inflammatory cytokines in a way that indicates that yoghurts from both ovine and bovine milk exhibit mostly anti-inflammatory properties.

### 3.5. Principal Component Analysis

**Figure 3:**
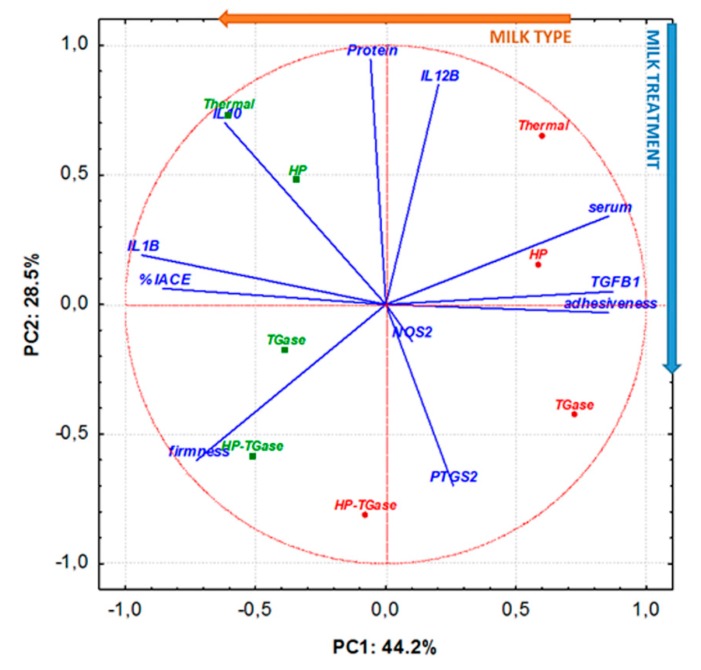
Principal component analysis (PCA) biplot for the investigation of the effect of milk type and milk treatment on the quality and bio-functional properties of yoghurt samples prepared from bovine (red cycles) or ovine (green squares) milk (the codes Thermal, TGase, HP, and HP–TGase represent the treatment of milk).

In order to further evaluate the effect of the milk type and milk treatment on the quality and bio-functional properties of yoghurt samples, the data obtained from the physicochemical (pH, serum, protein), textural (firmness, adhesiveness) and bio-functional (*%IACE*, *IL1B*, *IL12B*, *PTGS2*, *IL10*, *TGFB1*, *NOS2*) attributes of the prepared yoghurt samples at D+3 were submitted to principal component analysis (PCA). According to the PCA biplot (Figure 3), principal components (PC) 1 and 2 accounted together for 72.7% of the total explained variance.

The capability of coagulum to retain water (serum), adhesiveness and *TGFB1* gene expression was positively correlated with PC1, whereas firmness, anti-hypertensive activity (*%IACE*) and *IL1B* gene expression were negatively correlated with the same principle component. Moreover, the gene expression of *PTGS2* was negatively correlated with PC2, whereas protein content, and *IL12B* and *IL10* gene expression were positively correlated with PC2. With regard to *NOS2* gene expression, according to the estimated table of factor coordinates of variables, it was found to be strongly and negatively correlated with principal component 4 (representing 8.3% of total variance). Moreover, it was observed that milk type was correlated to PC2, whereas milk treatment was strongly correlated to PC1 (as shown by vectors).

From the PCA biplot, it is obvious that milk type and the kind of applied treatment in milk affect the quality profile of yoghurt, resulting in a clear classification of bovine and ovine yoghurt samples into two main groups, with the use of ovine milk promoting the production of samples with improved firmness, enhanced anti-hypertensive activity and immunomodulatory properties. Additionally, according to the sample distribution, it is evident that differentiations in milk treatment led to the production of yoghurt samples with completely different characteristics. Consequently, the implementation of the TGase and/or HP–TGase treatment of milk led to the production of acid gels with reduced whey separation, improved firmness and enhanced bio-functional characteristics, whereas the individual application of HP in milk led to products with similar technological characteristics and slightly enhanced bio-functional characteristics compared to those of samples from thermally treated milk.

## 4. Conclusions

Modern consumer demands are related not only to high nutritional value foods but also foods with functional properties. These foods include yoghurt produced with alternative technologies, such as the high pressure and transglutaminase treatment of milk and milk proteins, respectively. Moreover, yoghurt made from both bovine and—especially—ovine milk is known for its functional properties. Previous studies have shown that the application of HP and/or TGase treatment of milk during yoghurt manufacturing could be more inexpensive compared to the addition of external protein sources in the pursuit of the formation of a stable and compact acid coagulum and to overcome the common problems of non-fat or low-fat dairy products (e.g., weak texture, phase separation, sensorial defects, etc.). This study revealed that, apart from the improvement of the quality characteristics of yoghurt, these technologies led to the maintenance or even improvement of the bio-functional profile of either bovine or ovine yoghurt. More specifically, yoghurt prepared from bovine HP–TGase-treated and ovine HP-treated milk exhibited the greatest ACE-inhibitory activity, even after 42 days of storage. In addition, yoghurt prepared from TGase-treated milk exhibited the best results in eliciting the same levels of expression of important anti-inflammatory genes, such as TGFB1, while at the same time leading to reduced levels of the expression of pro-inflammatory genes, such as IL1B and IL12B from monocytes. With regard to milk type, it was observed that yoghurt produced from ovine milk had higher anti-hypertensive and immunomodulatory properties than yoghurt from bovine milk, regardless of the applied treatment in milk. Nevertheless, further research needs to be performed in specific peptidic fractions with anti-hypertensive activity and in simple systems of different types of milk in order to identify the mechanisms of the effect of HP and TGase treatment of milk on the IACE and immunomodulatory activity of fermented dairy products.

## Figures and Tables

**Table 1:** List of the experimental milk samples used.

Code	Type of Milk	Performed Treatment for Milk Sample
Thermal	Bovine or Ovine	Thermal treatment at 95 °C for 5 min
TGase		Thermal treatment at 95 °C for 5 min, followed by TGase treatment at 43 °C for 180 min and inactivation of the enzyme at 80 °C for 1 min
HP		HP treatment at 600 MPa and 55 °C for 10 min
HP–TGase		HP treatment at 600 MPa and 55 °C for 10 min, followed by TGase treatment at 43 °C for 180 min and inactivation of the enzyme at 80 °C for 1 min

TGase: transglutaminase, HP: high pressure.

**Table 2:** Fermentation kinetics parameters and fermentation times of bovine and ovine milk samples.

	Lag Phase *λ* (min)	Maximum Acidification Rate *μ* (pH/min)	*R^2^*	Fermentation Time (min)
**Bovine**				
Thermal	116 ^f^ (± 1.1)	0.0181 ^b^ (± 0.0004)	0.999	220 ^bc^ (± 15)
TGase	90.4 ^b^ (± 0.6)	0.0190 ^cd^ (± 0.0012)	0.998	190 ^a^ (± 10)
HP	123 ^g^ (± 0.0)	0.0198 ^d^ (± 0.0000)	0.996	230 ^cd^ (± 0.0)
HP–TGase	107 ^e^ (± 0.0)	0.0185 ^bc^ (± 0.0000)	0.998	210 ^b^ (± 5.0)
**Ovine**				
Thermal	93.2 ^c^ (± 1.2)	0.0151 ^a^ (± 0.0002)	0.992	260 ^e^ (± 5.0)
TGase	93.3 ^c^ (± 0.1)	0.0147 ^a^ (± 0.0001)	0.995	240 ^d^ (± 0.0)
HP	103 ^d^ (± 0.2)	0.0196 ^cd^ (± 0.0001)	0.997	210 ^b^ (± 0.0)
HP–TGase	88.1^a^ (± 1.0)	0.0178 ^b^ (± 0.0001)	0.998	210 ^b^ (± 5.0)

Values are means (± stdev) of three measurements of two independent experiments (n = 6). Different letters among lines in the same column indicate significant difference (*p* < 0.05) between tested samples according to Duncan’s mean values post hoc comparison test.

**Table 3:** Physicochemical characteristics and main textural attributes of bovine and ovine yoghurt samples as evaluated after 3 (D+3) days of their production.

	Physicochemical Characteristics			Main Textural Attributes		
	pH	% Lactic Acid	Serum (g/100g Product)	Firmness (g)	Adhesiveness (g·s)	Cohesiveness *
**Bovine**						
Thermal	4.48 ^e^ (± 0.01)	0.79 ^b^ (± 0.01)	49.8 ^f^ (± 1.7)	74.3 ^a^ (± 3.8)	38.1 ^ef^ (± 1.0)	0.52 ^b^ (± 0.01)
TGase	4.44 ^d^ (± 0.02)	0.79 ^b^ (± 0.01)	39.6 ^e^ (± 1.4)	146 ^b^ (± 2.5)	34.3 ^f^ (± 2.7)	0.53 ^bc^ (± 0.01)
HP	4.51 ^ef^ (± 0.04)	0.98 ^e^ (± 0.00)	47.5 ^f^ (± 0.6)	89.0 ^a^ (± 6.0)	44.6 ^e^ (± 4.7)	0.52 ^b^ (± 0.02)
HP–TGase	4.18 ^a^ (± 0.01)	0.96 ^e^ (± 0.02)	32.9 ^d^ (± 2.9)	333 ^d^ (± 6.7)	121 ^b^ (± 8.9)	0.44 ^a^ (± 0.01)
**Ovine**						
Thermal	4.42 ^cd^ (± 0.02)	0.82 ^bc^ (± 0.03)	23.8 ^c^ (± 0.8)	220 ^c^ (± 6.8)	152 ^a^ (± 3.7)	0.51 ^b^ (± 0.02)
TGase	4.54 ^f^ (± 0.02)	0.72 ^a^ (± 0.01)	13.8 ^b^ (± 1.0)	338 ^d^ (± 14)	80.1 ^d^ (± 0.4)	0.60 ^d^ (± 0.01)
HP	4.40 ^bc^ (± 0.01)	0.86 ^cd^ (± 0.02)	32.2 ^d^ (± 1.1)	219 ^c^ (± 2.8)	96.1 ^c^ (± 2.3)	0.55 ^c^ (± 0.00)
HP–TGase	4.37 ^b^ (± 0.02)	0.88^d^ (± 0.05)	8.76 ^a^ (± 0.9)	548 ^e^ (± 18)	94.3 ^c^ (± 0.2)	0.62 ^d^ (± 0.01)

* Dimensionless. Values are means (± standard deviation) of three measurements of two independent experiments (n = 6). Different letters among lines in the same column indicate significant differences (*p* < 0.05) between tested samples according to Duncan’s multiple range test.

**Table 4:** Immunomodulatory properties of bovine and ovine yoghurt WSEs as evaluated though the expression of pro-inflammatory and anti-inflammatory genes by ovine monocytes, and analyzed after 3 (D+3) and 42 (D+42) days of yoghurt sample production.

	IL1B	IL10	IL12B	PTGS2	NOS2	TGFB1
**Bovine**						
**D+3**						
Thermal	0.952 ^abc^ (± 0.027)	2.428 ^b^ (± 0.051)	0.562 ^a^ (± 0.066)	1.690 ^ab ^(± 0.033)	4.150 ^cd ^(± 0.583)	2.133 ^def ^(± 0.226)
TGase	0.972 ^abc^ (± 0.090)	1.460 ^a^ (± 0.339)	0.312 ^a^ (± 0.051)	1.884 ^ab ^(± 0.341)	3.106 ^ab ^(± 0.907)	2.291 ^ef ^(± 0.150)
HP	0.815 ^ab^ (± 0.072)	1.441 ^a^ (± 0.213)	0.501 ^a^ (± 0.143)	1.430 ^a ^(± 0.146)	2.862^a ^(± 0.088)	1.900 ^bcde ^(± 0.209)
HP–TGase	1.159 ^c ^(± 0.074)	1.427 ^a^ (± 0.216)	0.366 ^a^ (± 0.089)	2.481 ^bc^ (± 0.112)	3.139 ^ab ^(± 0.743)	1.574 ^abc ^(± 0.286)
**D+42**						
Thermal	0.772 ^a ^(± 0.088)	1.446 ^a^ (± 0.093)	0.478 ^a^ (± 0.132)	1.330 ^a ^(± 0.093)	2.724 ^a ^(± 0.436)	1.621 ^abc ^(± 0.141)
TGase	1.096 ^abc^ (± 0.176)	1.359 ^a^ (± 0.151)	0.318 ^a^ (± 0.019)	1.421 ^a ^(± 0.142)	3.210 ^ab ^(± 0.294)	1.735 ^abcd ^(± 0.236)
HP	1.046 ^abc^ (± 0.064)	1.495 ^a^ (± 0.118)	0.521 ^a^ (± 0.121)	1.360 ^a ^(± 0.051)	2.995 ^a ^(± 0.144)	1.436 ^a ^(± 0.442)
HP–TGase	1.111 ^bc ^(± 0.064)	1.242 ^a^ (± 0.181)	0.319 ^a^ (± 0.080)	1.637 ^ab^ (± 0.256)	4.093 ^cd ^(± 0.643)	1.545 ^ab ^(± 0.081)
**Ovine**						
**D+3**						
Thermal	2.319 ^f ^(± 0.217)	3.052 ^c^ (± 0.326)	2.366 ^c^ (± 0.032)	3.073 ^cd ^(± 0.353)	5.508 ^efg ^(± 0.638)	2.448 ^fg ^(± 0.058)
TGase	1.896 ^e ^(± 0.228)	2.404 ^b^ (± 0.463)	1.640 ^b^ (± 0.043)	3.358 ^d ^(± 0.559)	5.272 ^ef ^(± 0.440)	1.978 ^cde ^(± 0.009)
HP	2.109 ^ef ^(± 0.238)	2.491 ^b^ (± 0.536)	3.253 ^d^ (± 0.235)	4.964 ^e ^(± 0.387)	3.448 ^abc ^(± 0.378)	3.058 ^h ^(± 0.279)
HP–TGase	2.130 ^ef ^(± 0.324)	2.132 ^b^ (± 0.194)	1.888 ^b^ (± 0.095)	3.503 ^d ^(± 0.590)	5.451 ^efg ^(± 0.358)	2.412 ^f ^(± 0.382)
**D+42**						
Thermal	3.085 ^g ^(± 0.259)	3.176^c^ (± 0.366)	5.912 ^e^ (± 0.564)	4.754 ^e ^(± 0.180)	6.182 ^g ^(± 0.100)	3.820 ^j ^(± 0.114)
TGase	1.481 ^d ^(± 0.251)	1.409 ^a^ (± 0.028)	2.480 ^c^ (± 0.291)	2.111 ^ab ^(± 0.098)	4.708 ^de ^(± 0.009)	1.668 ^abc ^(± 0.033)
HP	2.123 ^ef ^(± 0.176)	2.512 ^b^ (± 0.106)	3.489 ^d^ (± 0.336)	3.588 ^d ^(± 0.640)	5.785 ^fg ^(± 0.292)	2.515 ^fg ^(± 0.198)
HP–TGase	2.057 ^ef ^(± 0.078)	3.231 ^c^ (± 0.140)	5.979 ^e^ (± 0.556)	4.455 ^e ^(± 0.104)	3.922 ^bcd ^(± 0.302)	2.815 ^gh ^(± 0.163)
Milk Type (MT)	***	***	***	***	***	***
Milk Treatment (MT)	***	***	***	***	***	***
Storage Time (ST)	n.s.	n.s.	***	n.s.	n.s.	n.s.
MO × MT × ST	***	***	***	***	***	***

IL1B: interleukin 1; IL10: interleukin 10, IL12B: interleukin 12; PTGS2: cyclooxygenase-2; NOS2: nitric oxide synthetase; TGFB1: transforming growth factor beta 1. Values are means (± standard deviation) of three measurements of two independent experiments (n = 6). Different letters among lines of the same column indicate significant differences (*p* < 0.05) between tested samples according to Duncan’s multiple range test. Symbols represent level of significance, where ***: *p* < 0.001, n.s.: not significant.
